# Burden of cardiovascular disease attributed to air pollution: a systematic review

**DOI:** 10.1186/s12992-024-01040-0

**Published:** 2024-05-03

**Authors:** Amir Hossein Khoshakhlagh, Mahdiyeh Mohammadzadeh, Agnieszka Gruszecka-Kosowska, Evangelos Oikonomou

**Affiliations:** 1 Department of Occupational Health Engineering, School of Health, Kashan University of Medical Sciences, Kashan, Iran; 2 Department of Health in Emergencies and Disasters, School of Public Health, Tehran University of Medical Sciences, Tehran, Iran; 3 Climate Change and Health Research Center (CCHRC), Institute for Environmental Research (IER), Tehran University of Medical Sciences, Tehran, Iran; 4 AGH University of Krakow, Faculty of Geology, Geophysics and Environmental Protection, Department of Environmental Protection, al. A. Mickiewicza 30, 30-059 Krakow, Poland; 5 Department of Cardiology, ‘Sotiria’ General Hospital, National and Kapodistrian University of Athens, School of Medicine, Athens, Greece

**Keywords:** Air pollution, Burden of disease, Cardiovascular disease, Disability, Mortality

## Abstract

**Background:**

Cardiovascular diseases (CVDs) are estimated to be the leading cause of global death. Air pollution is the biggest environmental threat to public health worldwide. It is considered a potentially modifiable environmental risk factor for CVDs because it can be prevented by adopting the right national and international policies. The present study was conducted to synthesize the results of existing studies on the burden of CVDs attributed to air pollution, namely prevalence, hospitalization, disability, mortality, and cost characteristics.

**Methods:**

A systematic search was performed in the Scopus, PubMed, and Web of Science databases to identify studies, without time limitations, up to June 13, 2023. Exclusion criteria included prenatal exposure, exposure to indoor air pollution, review studies, conferences, books, letters to editors, and animal and laboratory studies. The quality of the articles was evaluated based on the Agency for Healthcare Research and Quality Assessment Form, the Newcastle–Ottawa Scale, and Drummond Criteria using a self-established scale. The articles that achieved categories A and B were included in the study.

**Results:**

Of the 566 studies obtained, based on the inclusion/exclusion criteria, 92 studies were defined as eligible in the present systematic review. The results of these investigations supported that chronic exposure to various concentrations of air pollutants, increased the prevalence, hospitalization, disability, mortality, and costs of CVDs attributed to air pollution, even at relatively low levels. According to the results, the main pollutant investigated closely associated with hypertension was PM_2.5_. Furthermore, the global DALY related to stroke during 2016–2019 has increased by 1.8 times and hospitalization related to CVDs in 2023 has increased by 8.5 times compared to 2014.

**Conclusion:**

Ambient air pollution is an underestimated but significant and modifiable contributor to CVDs burden and public health costs. This should not only be considered an environmental problem but also as an important risk factor for a significant increase in CVD cases and mortality. The findings of the systematic review highlighted the opportunity to apply more preventive measures in the public health sector to reduce the footprint of CVDs in human society.

**Supplementary Information:**

The online version contains supplementary material available at 10.1186/s12992-024-01040-0.

## Introduction

Cardiovascular diseases (CVDs) are responsible for most of the deaths and disabilities worldwide [1, 2]. In 2017, CVDs resulted in more than 360 million disability-adjusted life years (DALYs) (Table 1) worldwide, making it a significant health concern in both developed and developing countries [3, 4]. The World Health Organization (WHO) reports that CVDs, including ischemic heart disease (IHD), atrial fibrillation (AF), stroke, heart failure (HF), and other cardiovascular disorders account for 43% of all deaths from non-communicable diseases (NCDs) [5].

**Table 1:** Glossary of terms

**DALY:** Disability-adjusted life years A DALY refers to the loss of one year of full health in a person’s life due to injury, disease, or disability. It is a measure used to quantify the burden of disease and to compare the overall health and life expectancy of different populations. DALY = YLL + YLD
**YLL:** Years of life lost to premature death YLLs are calculated by multiplying the number of deaths by a global standard life expectancy at the age of death. It is a measure of premature mortality that takes into account both the frequency of deaths and the age at which it occurs
**YLD:** Years of life lived with disability Each YLD represents one full year of healthy life lost due to illness or disability
**Incidence Rate** It is the proportion of new cases within a specific time period to the total number of people at risk for the disease. The total observation time of the population within a given calendar year refers to the approximate number of people in the population at the beginning of the year
**Mortality Rate**: Deaths in the population It is the number of deaths from a disease divided by the total population. The total observation time of the population within a given calendar year refers to the approximate number of people in the population at the beginning of the year

Currently, research revealed that more than 80% of CVD cases can be prevented by addressing risk factors such as smoking, arterial hypertension, diabetes mellitus, hypercholesterolemia, overweight, lack of physical activity, unhealthy diet, and exposure to air pollution [6, 7]. Despite the significant impact of environmental factors, especially air pollution, on health outcomes, they are often overlooked in the assessment of the global burden of disease (GBD) [8].

Air pollution is a major environmental concern in terms of the occurrence of adverse health effects and the negative impact on public health [9]. Fossil fuel consumption, especially in industries and transportation, is considered one of the most important sources of air pollution after the industrial revolution. In addition to being the main perpetrator of hazardous pollutant emissions, industry also plays an undeniable role in the increase in the average temperature of the Earth [10]. The expotential increase in industrialization results in a devastating impact on the environment. In some countries with a high Human Development Index (HDI), this leads to their largest share in the world’s greenhouse gases and hazardous pollutant emissions.

As a consequence, preventive policies and tax measures were introduced, particularly for these activities with high emission levels. Unfortunately, the existing global disparities caused an enormous difference in the rate of use of clean fuels. Modern renewable energy sources supply only 2.3% of electricity in low HDI countries, whereas this figure is 11% in countries with very high HDI. The dependance on the use of biomass fuel as an energy source was equal to 92% of households in countries with low HDI compared to 7.5% in countries with very high HDI. This led to the failure to limit the consumption of fossil fuels despite the efforts made [11–13]. Biomass fuel is used for heating, cooking, and providing lighting inside the house, as well as an energy source for occupational, industry, and transportation purposes, which can cause the release of high levels of air pollutants. According to studies, air pollution is considered as a consequence of population growth and urbanization, which is considered an important factor in premature mortality. This in turn, increases the costs of many NCDs, especially among local populations [14–16].

According to the lancet commission on pollution and health, harmful environmental conditions are responsible for approximately 9 million excess deaths worldwide, half of them attributed to air pollution [8]. The monetary costs of premature deaths attributed to air pollution in 2020 were estimated at 2.2 trillion dollars, which was equivalent to 2.4% of the gross world product (GWP) [12]. Additionally, two-thirds of the health effects caused by exposure to air pollutants were found to be related to cardiovascular mortality and other health complications [17]. Specifically, acute myocardial infarction (AMI) and stroke contributed to almost 50% of these adverse effects, resulting in a significant burden on healthcare costs worldwide [17, 18].

The published data report of the WHO indicated that almost 99% of the global population is exposed to inhalation of air pollutants that exceed the air quality threshold values recommended by this institution [19]. This alarming statistic revealed how much impact air pollution could have on the increase in CVDs, hospitalizations, disability, cases of mortality, and increase in economic costs.

The increase in the publication of related studies during the last 10 years can effectively draw a risk perspective of the growth in the burden of CVDs caused by exposure to air pollution. The results of these investigations may provide important evidence for implementing air pollution control measures based on maintaining the input-output balance (policy cost-benefit), especially in developing countries.

However, the existence of some limitations and gaps restricts the generalization of the results of these researches, including conducting each study in a limited number of countries [20–22], investigating the effect of air pollution on only one type of CVDs [23–25], examining a limited number of air pollutants [25–27], participants of only one gender (male or female) [28–30] and subjects with a relatively high socio-economic status [30]. In addition, considering that no review study has been published in this field, it seems necessary to conduct a systematic review to retrieve related studies and cover the gaps mentioned above to achieve more comprehensive results.

This systematic review gathers and summarizes up-to-date studies on the burden of CVDs, including prevalence, disability, hospitalization, mortality, and cost, caused by exposure to air pollution.

## Methods

### Research protocol

This systematic review was registered in the International Prospective Register of Systematic Reviews (PROSPERO, CRD42023434702) and adhered to the PRISMA (Preferred Reporting Items for Systematic Review and Meta-analyses) statement.

### Search strategy and data screening

The search was carried out without timeframe, up to June 13, 2023. A systematic search of the databases Scopus, PubMed, and Web of Science was conducted using the following keywords:

- **Disease:** “Cardiovascular Disease*”, “Myocardial Infarction”, “Heart Failure”, Hypertension, Myocarditis, Arrhythmia, “Coronary Heart Disease*”, “Cerebrovascular Disease*”, “Abnormal Heart Rhythms”, “Aorta Disease*”, “Heart Attack”, “Coronary Artery Disease*”, Cardiomyopathy, “Heart Muscle Disease*”, “Pericardial Disease*”, “Peripheral Vascular Disease*”, Stroke, “Vascular disease*”, Angina, “Rheumatic Heart Disease*”;
- **Disease burden**: “Illness Cost*”, “Sickness Cost*”, “Illness Burden*”, “Disease Burden*”, “Disease Cost*”, “Economic Burden of Disease”, “Disability-Adjusted Living Years”, DALY, Mortality, Morbidity, “Years of Life Lost”, YLL, “Years Lost due to Disability”, YLD;
- **Exposure:** “Air pollution”.

Two researchers, M.M. and A.H.Kh., extracted keywords and conducted a systematic search for Title/Abstract and Mesh (if any). Studies obtained from databases were integrated using EndNote X20 software. After removing duplicates, M.M. and A.H.Kh. independently screened and extracted the studies. The third author (E.O.) resolved any ambiguities or contradictions during the review process. To ensure that no eligible studies were missed, the reference list of the selected studies was systematically searched. Additionally, a hand search was also conducted in parallel.

### Entry and exit criteria of the study

In this systematic review, studies focused on prenatal exposure to air pollution and the impact of indoor air pollution on the burden of CVDs were excluded. Furthermore, review studies, conference studies, books, letters to the editors, and animal/laboratory studies were omitted and only original articles published in English and peer reviewed were examined.

### Extracting the data

After reviewing and selecting eligible studies, their results were summarized in an electronic form in the Excel 2016 software. The data sheet encompassed various details such as author names, year of publication, title, country of investigation, number of participants, age range, gender, and type of pollutant. Variables related to disease burden included prevalence, hospitalization, disability (measured in disability-adjusted life years DALY), years lost to disability (YLD), years of life lost (YLL), mortality (mortality rate and death), and cost (total cost, economic loss due to missed work days, and overall economic losses).

### Quality control

Two researchers, M.M. and A.H.Kh., assessed the quality of selected studies using a self-established scale. This scale was based on the Agency for Healthcare Research and Quality Assessment Form, the Newcastle–Ottawa Scale [31], and the Drummond Criteria [32]. Based on this method, the quality of studies is determined by answering 9 questions, that are presented in Table 2. Questions 1–8 can only be answered as “yes” (1 score) or “no” (0 score). Question 9 can be answered as: “yes” (2 scores), “likely” (1 score), and “no” (0 score). The scores obtained were combined after confirmation, and each study was classified based on its quality score (between 0 and 10 points) into one of three categories: A, B, or C. A study with a quality score equal to or above 8 received category A. If the quality score was between 4 and 7, the study received category B. If the quality score was less than 4 a study received category C [33]. Only articles classified in categories A and B were included in the systematic review analysis.

**Table 2:** Quality assessment scale [33]

1.	Was the objective clear?
2.	Was the data source official?
3.	Was the study population-based?
4.	Was the sampling randomized?
5.	Was the study region nationwide?
6.	Were the diagnostic criteria clear?
7.	Were the results comparatively analyzed?
8.	Did the outcome indicators include DALY/YLL/YLD?
9.	Can the results be externalized?

All items had two possible answers (except item 9): yes (+) and no (-). Item 9 had three possible responses: yes (++), likely (+), and no (-)

*DALY* Disability-adjusted living years, *YLL* Years of life lost, *YLD* Years lost due to disability

### Study selection

**Fig. 1:**
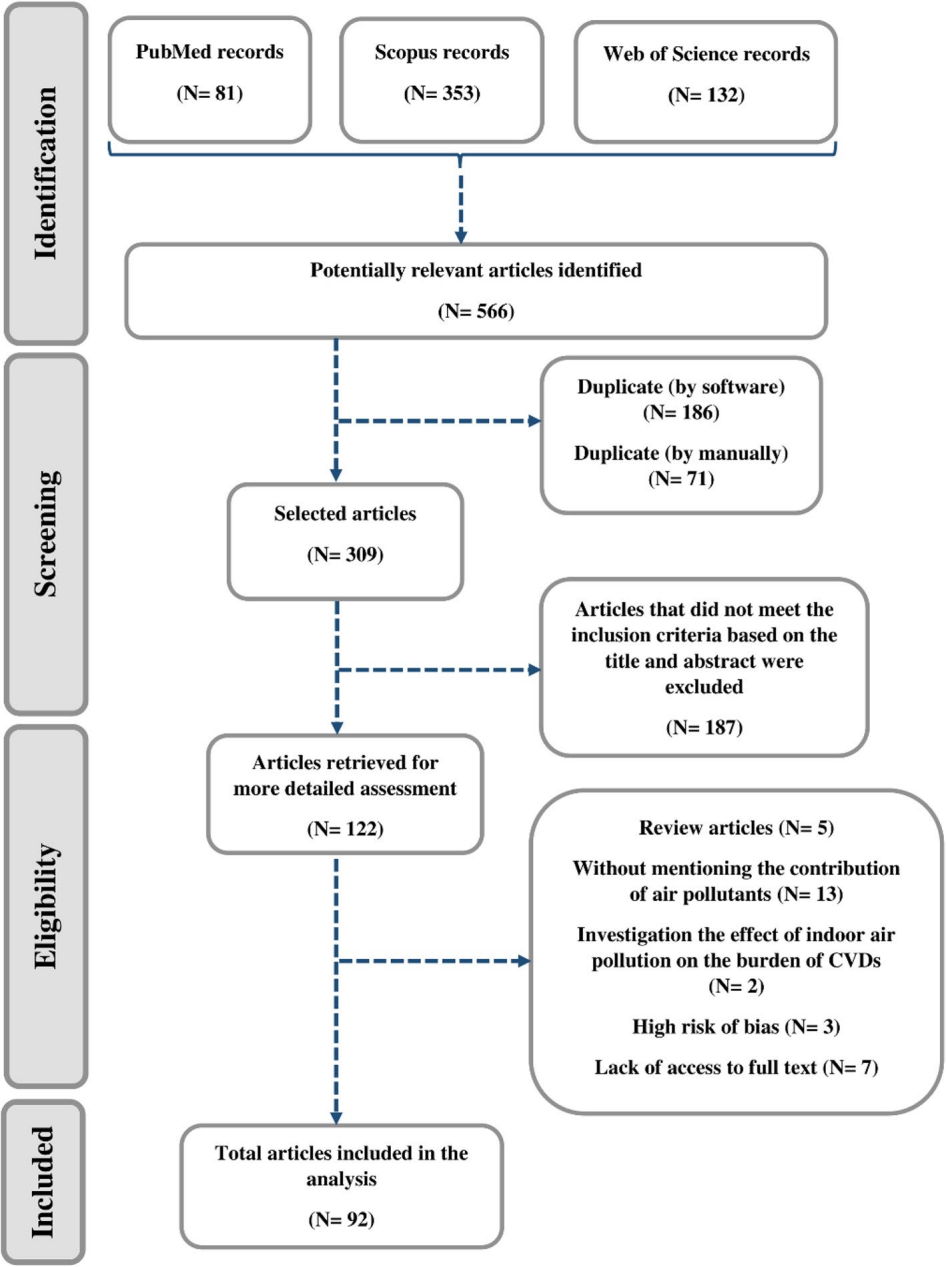
PRISMA flow diagram of the literature search on CVDs related to air pollution

A systematic search was carried out in the PubMed, Scopus, and Web of Science databases as presented in Fig. 1. The 566 articles found were screened using EndNote X20 software based on title and abstract. Then 122 articles remained, and due to the inaccessibility of the full text of 7 studies, 115 full texts were thoroughly examined based on inclusion/exclusion criteria and quality assessment. Finally, 92 studies were defined eligible in this systematic review.

### Synthesis of results

In this study, we found various study designs that caused differences in methodology and context that made it unsuitable to perform a quantitative synthesis or meta-analysis. Therefore, we combined the study results in a narrative format, which included information about the type of pollutant, its mean concentration, and the disease burden variables, including prevalence (Table 3), hospitalization (Table 4), disability (disability-adjusted living years DALY, years lost due to disability YLD, and years of life lost YLL) (Supplementary Material 1), mortality (mortality rate, death) (Supplementary Material 2), and costs (total cost, economic loss from loss of the work day, and economic losses) (Table 5).

**Table 3:** Prevalence of cardiovascular diseases (CVDs) attributed to air pollution

**Author (year)**	**Title**	**Country (city)**	**Number of samples**	**Gender**	**Age (years)**	**Pollutant**	**Mean concentration (μg/m**^**3**^**)**	**Prevalence (%)**
Peng Yin (2017) [28, 34]	Long-term Fine Particulate Matter Exposure and Non accidental and Cause-specific Mortality in a Large National Cohort of Chinese Men	China (45 districts/ counties)	189,793	Male	54.8	PM_2.5_	43.7	Hypertension: 26.9%
Dorairaj Prabhakaran (2018) [7]	The changing patterns of cardiovascular diseases and their risk factors in the states of India: The Global Burden of Disease Study 1990–2016	India (national)	Nd	Male-female	≥ 10	PM_2.5_	Nd	Hypertension: 21.1%
Alireza Khajavi (2019) [20]	Impact of temperature and air pollution on cardiovascular disease and death in Iran: A 15-year follow-up of Tehran Lipid and Glucose Study	Iran (Tehran)	9,731	Male–female	47.7	CO, O_3_, PM_2.5_, PM_10_, NO_2_, SO_2_	Nd	CVD: 13.9%
Jing Xu (2020) [29]	Airborne Metals Exposure and Risk of Hypertension in the Sister Study	United States	47,595	Female	35–74	Arsenic Cadmium Chrome Cobalt Lead Manganese Mercury Nickel Selenium Antimony	**Median:** 4.36 × 10^−4^ 5.06 × 10^−4^ 1.67 × 10^−3^ 9.35 × 10^−4^ 5.73 × 10^−4^ 7.54 × 10^−5^ 9.71 × 10^−6^ 3.11 × 10^−5^ 8.51 × 10^−5^ 9.11 × 10^−6^	Hypertension: 33%
Xueli Yang (2020) [26]	Associations of long-term exposure to ambient PM_2.5_ with mortality in Chinese adults: A pooled analysis of cohorts in the China-PAR project	China (national)	116,821	Male–female	51.6	PM_2.5_	64.9	Hypertension: 31.8%
Tiantian Li (2020) [35]	Cohort profile: Sub-clinical outcomes of polluted air in China (SCOPA-China cohort)	China (Heilongjiang, Sichuan, Shandong, Guangdong, Jiangsu, Hubei, Shaanxi, Hebei, Hunan, Guangxi, Zhejiang, Beijing, Tianjin, Henan, Shanxi)	Nd	Male-female	64	PM_2.5_ PM_10_ O_3_ SO_2_ NO_2_ CO	PM_2.5_ = 48.3 PM_10_ = 82.5 O_3_ = 54.1 SO_2_ = 18.0 NO_2_ = 37.1 CO = 0.967	Hypertension: 35.4% CHD: 9.00% Stroke: 3.20% Arrhythmia: 3.20%
Frauke Hennig (2020) [36]	Air Pollution and Progression of Atherosclerosis in Different Vessel Beds - Results from a Prospective Cohort Study in the Ruhr Area, Germany	Germany (Mülheim, Essen, Bochum)	4,814	Male-female	58.9–59.1	PM_2.5_ PM_10_ NO_2_	PM_2.5_ = 20.3 PM_10_ = 16.7 NO_2_ = 39.5	Hypertension: 53% Developed coronary heart disease: 0.5–2.4%
Min Zhao (2020) [37]	A Global Analysis of Associations between Fine Particle Air Pollution and Cardiovascular Risk Factors: Feasibility Study on Data Linkage	Global (10 countries)	Nd	Male-female	64.9	PM_2.5_	38.1	Hypertension: 74.5%
Dwi Agustian (2020) [22]	Feasibility of Indonesia Family Life Survey Wave 5 (IFLS5) Data for Air Pollution Exposure–Response Study in Indonesia	Indonesia (national)	58,304	Male-female	< 10– ≥ 25	NO_2_ O_3_ PM_10_ PM_2.5_	Nd	Hypertension: 14.8% Stroke: 1.2% Heart Problem:2.0%
Markey Johnson (2020) [25]	Traffic-Related Air Pollution and Carotid Plaque Burden in a Canadian City with Low-Level Ambient Pollution	Canada (London)	2,227	Male-female	62.9	NO_2_	10.1	Hypertension: 69%
Seyed M. Karimi (2020) [38]	Continuous Exposure to Ambient Air Pollution and Chronic Diseases: Prevalence, Burden, and Economic Costs	Iran (Tehran)	67,049	Male-female	49.1–68.6	CO, NO_2_, O_3_, PM_10_	CO = 4,124.17 NO_2_ = 101.61 O_3_ = 42.9 PM_10_ = 101.7	Stroke: 1.4% Hypertension: 5.3%
Jiayue Xu (2021) [21]	Long-term effects of ambient PM_2.5_ on hypertension in multi-ethnic population from Sichuan province, China: a study based on 2013 and 2018 health service surveys	China (Sichuan)	31,462	Male-female	56	PM_2.5_	32.8	Hypertension: 5.6–15.9%
Jie Ban (2021) [27]	Associations between short-term exposure to PM_2.5_ and stroke incidence and mortality in China: A case-crossover study and estimation of the burden	China (10 counties)	Nd	Male-female	0–> 75	PM_2.5_	53.9	**[Per 10 μg/m**^**3**^** increase in PM**_**2.5**_** concentration]:** Stroke: 0.37% Ischemic stroke: 0.46% Hemorrhagic stroke: 0.13%
Evangelos Oikonomou (2021) [39]	The association of air pollutants exposure with subclinical inflammation and carotid atherosclerosis	Greece (Corinthia)	1,955	Male-female	≥ 40	CO, NO_2_, SO_2_	CO = 1.01–5.15 NO_2_ = 8.82–12.17 SO_2_ = 6.20–11.32	Hypertension: 69.0% CAD: 12.5% Carotid plaque: 22.3%
Jing Xu (2022) [30]	Fossil-fuel and combustion-related air pollution and hypertension in the Sister Study	United States (national)	47,467	Female	55.6	Biphenyl, Naphthalene, Polycyclic organic matter, Diesel emissions, 1,3-butadiene, Acetaldehyde, Benzene, Acrolein Formaldehyde, NO_2_	Diesel emissions = 0.48 Acetaldehyde = 1.76 Benzene = 0.88 Formaldehyde = 1.88 NO_2_ = 10.1 Biphenyl = 7.38 × 10^–5^ Naphthalene = 4.12 × 10^–2^ PAHPOM = 8.69 × 10^–3^ 1,3 butadiene = 5.61 × 10^–2^ Acrolein = 3.45 × 10^–2^	Hypertension: 34%
Qing Pan (2022) [40]	Identification of the susceptible subpopulations for wide pulse pressure under long-term exposure to ambient particulate matters	China (national)	69,215	Male-female	51.83	PM_1_, PM_2.5_, PM_10_	PM_1_ = 30.24 PM_2.5_ = 38.54 PM_10_ = 64.96	Wide pulse pressure: 15.8%
Hugo Grisales-Romero (2023) [14]	Local attributable burden disease to PM_2.5_ ambient air pollution in Medellín, Colombia, 2010–2016	Colombia (Medellín)	3,873	Male-female	0–≥ 80	PM_2.5_	35.8	IHD: 2.1% CEV: 2.3%
Shiyu Zhang (2023) [15]	Exposure to Air Pollution during Pre-Hypertension and Subsequent Hypertension, Cardiovascular Disease, and Death: A Trajectory Analysis of the UK Biobank Cohort	United Kingdom (national)	168,010	Male-female	54.2	PM_2.5_, PM_10_, NO_2_, NOx	PM_2.5_ = 9.95–10.06 PM_10_ = 19.23–19.41 NO_2_ = 29.00–29.93 NOx = 43.54–44.93	Hypertension: 8.18% CVD: 10.46%

*Nd* Not defined, *CVD* Cardiovascular Disease, *IHD* Ischemic Heart Disease, *CAD* Coronary Artery Disease, *CEV* Cerebrovascular Disease, *PM*_*1*_ Particulate matter smaller than 1 μm, *PM*_*2.5*_ Particulate matter smaller than 2.5 μm, *PM*_*10*_ Particulate matter smaller than 10 μm, *NO*_*2*_ Nitrogen dioxide, *NOx* Nitrogen oxides, *CO* Carbon monoxide, *SO*_*2*_ Sulphur dioxide, *O*_*3*_ Ozone

**Table 4:** Hospitalization due to cardiovascular diseases (CVDs) attributed to air pollution

**Author (year)**	**Title**	**Country (city)**	**Number of samples**	**Gender**	**Age (years)**	**Pollutant**	**Mean concentration (μg/m**^**3**^**)**	**Number of hospital admissions**^**a**^
Laura Anderko (2014) [41]	Identifying Populations at Risk: Interdisciplinary Environmental Climate Change Tracking	USA (District of Columbia)	2,773	Male-female	0–≥ 80	PM_2.5_, PM_10_, O_3_	PM_2.5_ = 20.9 PM_10_ = 11.8 O_3_ = 71.5	CVD: 58
Rakesh Ghosh (2016) [42]	Near-Roadway Air Pollution and Coronary Heart Disease: Burden of Disease and Potential Impact of a Greenhouse Gas Reduction Strategy in Southern California	USA (California)	1.2 million	Male-female	45–≥ 85	PM_2.5_, elemental carbon (EC)	Nd	CHD: 8.9 per 1,000 individuals
Gerardo Sanchez Martinez (2018) [43]	Health Impacts and Economic Costs of Air Pollution in the Metropolitan Area of Skopje	Republic of Macedonia (Skopje)	531,524	Male-female	≥ 30	PM_2.5_	49.2	CVD: 547
Chaicharn Pothirat (2019) [44]	Acute effects of air pollutants on daily mortality and hospitalizations due to cardiovascular and respiratory diseases	Thailand (Chiang Mai)	4,685	Male-female	Nd	PM_10_, PM_2.5_, SO_2_, NO_2_, CO, O_3_	Median PM_10_ = 37.36 PM_2.5_ = 20.42 SO_2_ = 2.62 NO_2_ = 27.92 CO = 1,130 O_3_ = 40.39	**Emergency visits:** Hypertension: 237 HF: 102 MI: 30 Stroke: 221 **Hospitalization:** Hypertension: 298 HF: 223 MI: 53 Stroke: 285
Xiaojuan Zhu (2019) [45]	Risks of hospital admissions from a spectrum of causes associated with particulate matter pollution	China (Chengdu)	3.72 million	Male-female	≤ 14–≥ 65	PM_2.5_, PM_10_	PM_2.5_ = 57.3 PM_10_ = 94.7	IHD: 32,117 MI: 2,228 Arrhythmias: 2,794 HF: 2,691 Stroke: 28,321 CEV: 44,260
Bangzhu Zhu (2019) [46]	Including intangible costs into the cost-of-illness approach: a method refinement illustrated based on the PM_2.5_ economic burden in China	China (76 cities)	5.67 million	Male-female	Nd	PM_2.5_	40–160	CVD: 106,888–128,116
Cheng-Pin Kuo (2021) [47]	Quantifying spatial heterogeneity of vulnerability to short-term PM_2.5_ exposure with data fusion framework	Taiwan (Tainan)	69,261	Male-female	< 65–≥ 65	PM_2.5_	25.12	CVD: 12,524
Yang Xie (2021) [48]	Short-Term Ambient Particulate Air Pollution and Hospitalization Expenditures of Cause-Specific Cardiorespiratory Diseases in China: A Multicity Analysis	China (74 cities)	88.5 million	Male-female	15–64–≥ 65	PM_2.5_	49.7	CHD: 54,600 Stroke: 23,989
Maria D. Castillo (2021) [49]	Estimating Intra-Urban Inequities in PM_2.5_-Attributable Health Impacts: A Case Study for Washington, DC	USA (Washington)	627,656	Male-female	0–99	PM_2.5_	10–17.1	IHD: 840 Stroke: 89
Marcos Lorran Paranhos Leão (2021) [50]	Health impact assessment of air pollutants during the COVID-19 pandemic in a Brazilian metropolis	Brazil (Recife)	1,653,461	Male-female	15–> 65	PM_10_, PM_2.5_	PM_10_ = 15.5–22 PM_2.5_ = 7.33–13	CVD: 11,188
Tao Liu (2022) [51]	Association of ambient PM_1_ with hospital admission and recurrence of stroke in China	China (292 cities)	1,006,798	Male-female	< 65–≥ 65	PM_1_	43.78	Ischemic stroke: 824,808 Transient ischemic attack: 62,535 Intracerebral hemorrhage: 83,693 Subarachnoid hemorrhage: 10,998
Wanyanhan Jiang (2022) [52]	The short-term effects and burden of particle air pollution on hospitalization for coronary heart disease: a time-stratified case-crossover study in Sichuan, China	China (9 cities)	104,779	Male-female	< 45–≥ 65	PM_10_, PM_2.5_	PM_10_ = 71.7 PM_2.5_ = 46.0	CCHD: 83,471 AMI: 12,817 UA: 3,946
Yuzhi Xi (2022) [53]	Association Between Long-term Ambient PM_2.5_ Exposure and Cardiovascular Outcomes Among US Hemodialysis Patients	United States (national)	314,079	Male-female	63.6	PM_2.5_	8.7	CVD: 208,113
Rodrigo Ugalde-Resano (2022) [54]	Short term exposure to ambient air pollutants and cardiovascular emergency department visits in Mexico City	Mexico (Mexico City)	48,891	Male-female	30–≥ 60	PM_10_, PM_2.5_, O_3_, NO_2_, SO_2_, CO	PM_10_ = 41.9 PM_2.5_ = 22.5 O_3_ = 116.4 NO_2_ = 50.3 SO_2_ = 10.6 CO = 0.98	**Daily visits:** Hypertension: 31 IHD: 4 CVD: 5
Teng-fei Dong (2023) [55]	Ambient Nitrogen Dioxide and Cardiovascular Diseases in Rural Regions: A Time-series Analyses Using Data from the New Rural Cooperative Medical Scheme in Fuyang, East China	China (Fuyang)	445,216	Male-female	18–≥ 75	NO_2_	36.3	CVDs: 488.2 IHD: 179.8 arrhythmias: 7.0 HF: 13.2 Ischemic stroke: 267.9 Hemorrhagic stroke: 20.2

*Nd* Not defined, *CVD* Cardiovascular Disease, *IHD* Ischemic Heart Disease, *HF* Heart Failure, *MI* Myocardial Infarction, *CAD* Coronary Artery Disease, *CEV* Cerebrovascular Disease, *CCHD* Chronic Coronary Heart Disease, *AMI* Acute Myocardial Infarction, *UA* Unstable Angina, *PM*_*1*_ Particulate matter smaller than 1 μm, *PM*_*2.5*_ Particulate matter smaller than 2.5 μm, *PM*_*10*_ Particulate matter smaller than 10 μm, *NO*_*2*_ Nitrogen dioxide, *NOx* Nitrogen oxides, *CO* Carbon monoxide, *SO*_*2*_ Sulphur dioxide, *O*_*3*_ Ozone

^a^Number of hospital admissions due to air pollution as reported in the analyzed studies

**Table 5:** Costs due to cardiovascular diseases (CVDs) attributed to air pollution

**Author (year)**	**Title**	**Country (city)**	**Number of samples**	**Gender**	**Age (years)**	**Pollutant**	**Mean concentration [μg/m**^**3**^**]**	**Total cost**	**Economic loss from lost workdays**	**Economic losses**
Bangzhu Zhu (2019) [46]	Including intangible costs into the cost-of-illness approach: a method refinement illustrated based on the PM_2.5_ economic burden in China	China (76 cities)	5.67 million	Male-female	Nd	PM_2.5_	40–160	Nd	Nd	CVD: 1.32 – 1.59 Billion CNY (US$ 182.3–219.6 million)^a^
Minghong Yao (2020) [56]	Estimating health burden and economic loss attributable to short-term exposure to multiple air pollutants in China	China (338 cities)	1.35 million	Male-female	Nd	PM_10_, SO_2_, NO_2_, CO, O_3_	PM_10_ = 79.73 SO_2_ = 18.14 NO_2_ = 30.23 CO = 0.96 O_3_ = 94.25	Nd	CVD: PM_10_: 319.66 Billion CNY (US$ 44.1 billion) SO_2_: 195.28 Billion CNY (US$ 26.9 billion) NO_2_: 604.02 Billion CNY (US$ 83.4 billion) CO: 440.43 Billion CNY (US$ 60.8 billion) O_3_: 496.24 Billion CNY (US$ 68.5 billion)	Nd
Yang Xie (2021) [48]	Short-Term Ambient Particulate Air Pollution and Hospitalization Expenditures of Cause-Specific Cardiorespiratory Diseases in China: A Multicity Analysis	China (74 cities)	88.5 million	Male-female	15–≥ 65	PM_2.5_	49.7	Nd	CHD: 103 million CNY (US$ 14.2 million) Stroke: 73 million CNY (US$ 10 million)	CHD: 458 million CNY (US$ 63.2 million) Stroke: 410 million CNY (US$ 56.6 million)
Wanyanhan Jiang (2022) [52]	The short-term effects and burden of particle air pollution on hospitalization for coronary heart disease: a time-stratified case-crossover study in Sichuan, China	China (9 cities)	104,779	Male-female	< 45–≥ 65	PM_10_, PM_2.5_	PM_10_ = 71.7 PM_2.5_ = 46.0	**CHD:** PM_10_ = 42.04 million CNY (US$ 5.8 million) PM_2.5_ = 67.25 million CNY (US$ 9.2 million)	Nd	Nd

*Nd* Not defined, *CVD* Cardiovascular Disease, *CHD* Coronary Heart Disease, *PM*_*1*_ Particulate matter smaller than 1 μm, *PM*_*2.5*_ Particulate matter smaller than 2.5 μm, *PM*_*10*_ Particulate matter smaller than 10 μm, *NO*_*2*_ Nitrogen dioxide, *NOx* Nitrogen oxides, *CO* Carbon monoxide, *SO*_*2*_ Sulphur dioxide, *O*_*3*_ Ozone, *CNY* Chinese yuan renminbi, *US$* USA dollar

^a^The conversion rate of CNY to US$ was calculated based on the daily rate in 2024

**Fig. 2:**
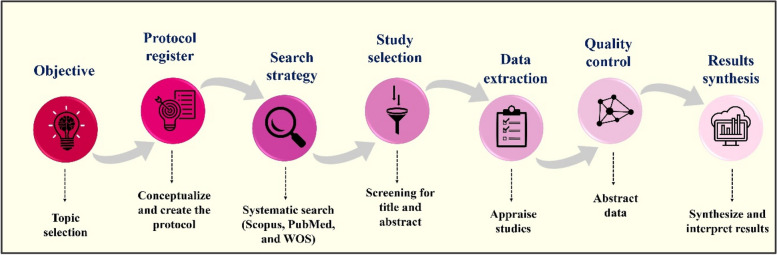
Visual representation of the complete framework of the systematic review guiding process

We followed a two-step process for narrative synthesis. In the first step, we classified the information into five separate tables according to the type of disease burden. In the second step, we evaluated the severity of the consequences by examining the relationship between exposure levels and disease burden. The brief visual scheme of the seven steps that comprises the complete systematic review framework performed in the study is presented in Fig. 2.

## Results

### Prevalence of CVDs attributed to air pollution

Hypertension is the most common disease associated with chronic exposure to air pollutants. As presented in Table 3 in this section, the main pollutants examined were PM_2.5_ (72.2%) and NO_2_ (50%), and were found to be closely associated with the incidence of hypertension according to the results obtained. The prevalence of CVDs related to air pollution was investigated in 18 studies from 10 countries around the world (Table 3) [7, 14, 15, 20–22, 25–30, 35–40]. Generally, 818,316 subjects of different age groups were evaluated during 2017–2023. A review of the listed studies showed that China was the most active in this field, publishing 6 studies [21, 26–28, 35, 40].

Based on the available information on the types of areas in the studies included in this systematic review, it was revealed that most of the investigations were carried out on a national and/or regional scale depending on the size of the country, for example, the United States [29, 30, 53, 57], Canada [58], Brazil [59], China [26, 35, 40, 60–66], and Europe [67–69]. There was also some research conducted worldwide [9, 37, 70–73], including several countries in international cohort studies.

Based on these studies, the prevalence of CVDs varied greatly, ranging from 0.5% for developed coronary heart disease to 74.5% for hypertension. Specifically, prevalence percentages for hypertension ranged from 5.3% to 74.5%, for coronary artery disease from 0.5% to 13.9%, for stroke from 1.2% to 3.2%, and for other CVDs from 2.00% to 1.46%. Reports of carotid plaque and arrhythmia were reported by a single study each. Consequently, the prevalence of carotid plaque related to air pollution was reported to be equal to 22.3% [15, 30, 38, 39], and the prevalence of arrhythmia was estimated to be 3.2% [36].

### Hospitalization due to CVDs attributed to air pollution

Table 4 presents a summary of the results of 15 studies [41–55] related to the hospitalization rate due to CVDs episodes as an adverse consequence of exposure to air pollution. Among the investigated pollutants, PM_2.5_ (86.6%) and PM_10_ (40%) were found to be the most common in the analyzed studies. According to the results obtained, 103,899,123 subjects from the following 7 countries were examined: China (6 studies) [45, 46, 48, 51, 52, 55], USA (4 studies) [41, 42, 49, 53], Taiwan (1 study) [47], Brazil (1 study) [50], Mexico (1 study) [54], Thailand (1 study) [44], and Republic of Macedonia (1 study) [43]. The publication years of these studies were from 2014 to 2023 and included all age groups.

Studies indicated that arrhythmias had the lowest hospital admission rate, with only 7 cases (0.001%) out of the 445,216 patients examined in China [55]. Also, Liu et al. [51] found that the hospitalization rate for ischemic stroke attributed to PM_1_ was 81.92% that was the highest rate among similar studies. This studies also demonstrated that an increase of 10 µg/m^3^ PM_1_ resulted in an increase of 0.53% (95% CI, 0.39%, 0.67%) in the hospital admission rate due to stroke [51].

### Disability due to CVDs attributed to air pollution

In the course of the investigations, 33 studies from 11 countries [7, 14, 23, 24, 38, 43, 59, 60, 62–64, 67–69, 74–86] and 6 studies based on worldwide data focused on the impact of air pollutants on global disability (DALY, YLD, YLL) in patients with CVDs [9, 70–73, 87]. China was the most active in this field by publishing 12 related studies. Among these, 21 studies specifically examined the role of PM_2.5_ in causing CVDs related disability during 2015–2023 (Supplementary Material 1). Generally, 2,338,344,120 subjects from different age groups were evaluated.

The results showed that over time, as industrial activity expanded and pollutant concentrations increased, the incidence of CVD-related disabilities, particularly stroke and IHD, also increased. The results of a global study showed that the DALY rate caused by exposure to PM_2.5_ in 1990 was 10 million years, which increased to 20 million years in 2019 (a two-fold increase). In addition, similar results were also observed in stroke [73]. Rueda et al. [85] also concluded in a national study in the Kingdom of Saudi Arabia that exposure to a concentration of 87.9 μg/m^3^ during 1990–2017 caused the increase of DALY rate 4 times due to IHD and 2.5 times due to stroke.

### Mortality due to CVDs attributed to air pollution

Supplementary Material 2 summarizes the results of 58 studies investigating the mortality rate of CVDs caused by air pollution [9, 14, 15, 20, 23, 26–28, 42, 43, 49, 50, 53, 56–69, 72–78, 80–82, 84–104]. These studies were carried out between 2015 and 2023 and have been published in 18 countries, including India, USA, Thailand, China, Canada, Republic of Macedonia, South Korea, Iran, Europe, Kazakhstan, Kingdom of Saudi Arabia, Brazil, Soviet Republics, Germany, Malaysia, Colombia, and the United Kingdom.

The researchers examined a total of 1,237,022,761 people in various age groups. In 48 different studies, researchers focused on PM_2.5_ as the main pollutant and its impact on deaths related to CVDs. The findings of these research confirmed that there is a direct correlation between the concentration of PM_2.5_ pollutants and the mortality rate associated with IHD and stroke.

Mazeli et al. [104] in a national study in Malaysia investigated the relationship between PM_2.5_ levels and CVD mortality. In this study, it was found that with the increase in pollutant concentration from 2000 (22 μg/m^3^) to 2013 (24 μg/m^3^), the death rate due to stroke has increased approximately twice, but this statistic remained almost constant in IHD [104]. In addition, the results of a recent study in Germany showed that exposure to levels of 13.7–10.8 μg/m^3^ PM_2.5_ can cause the death of 6,977 patients with IHD and 1,871 deaths due to stroke [86].

### Costs due to CVDs attributed to air pollution

The results obtained from 4 studies related to the economic burden caused by CVDs attributed to air pollution are shown in Table 5 [46, 48, 52, 56]. These studies were performed between 2019 and 2022, a total of 95,624,779 people from China were evaluated and PM_2.5_ was evaluated in all these four studies. Comparing the results of the studies showed that the greatest economic losses for CVD are related to PM_2.5_, as reported by Zhu et al. [46]. Furthermore, Yao et al. [56] reported the highest economic loss from lost workdays. This cost was calculated only for CVD attributed to PM_10_, SO_2_, NO_2_, CO, and O_3_. According to this study, the economic loss of CVD lost workdays attributed to NO_2_ was calculated at 604.02 billion CNY (US$ 83.4 billion), which was the highest amount compared to other pollutants [56]. According to Table 5, the total cost caused by CHD attributed to PM_2.5_ was found to be 1.6 times higher than those attributed to PM_10_ [52].

## Discussion

### Prevalence of CVDs attributed to air pollution

Hypertension was reported in studies to be the most prevalent among people who were chronically exposed to pollutants such as NO_2_, O_3_, PM_10_, PM_2.5_, and SO_2_. A 12-year follow-up study in the United Kingdom showed that exposure to air pollution was positively related to hypertension and its development in normotensive subjects [15]. This finding was consistent with a study by Prabhakaran et al. [7], which found an increasing trend in systolic blood pressure in Indian residents following an increase in pollutant concentration from 1990 to 2016. In the study of Karimi et al. [38] on the prevalence, burden, and economic costs of chronic diseases caused by air pollution in Tehran, Iran, the prevalence of hypertension was estimated at 5.3%. However, a German cohort study from 2020 found a prevalence of 53% for hypertension after exposure to PM_2.5_, PM_10_, and NO_2_ [36].

The results of an international cohort of Chinese men showed that exposure to an average level of 43.7 μg/m^3^ PM_2.5_ can increase the prevalence of hypertension to 26.9% [28]. This prevalence was consistent with the results reported by Prabhakaran et al. [7] (21.1%). In addition, Yang et al. [26] also demonstrated in a national cohort in China that exposure to a concentration of 64.9 μg/m^3^ of this pollutant can be associated with a prevalence of 31.8% of this health complication.

Although various studies revealed a positive correlation between exposure to air pollution and an increase in hypertension and subsequent consequences such as blindness, chest pain, pregnancy complications, heart attack, and stroke, the prevalence rate varied between different countries [15, 30, 38, 39]. Interestingly, developed countries had higher prevalence rates, contradicting the results of some studies [9, 60, 105]. The analysis of the research indicated that the studies conducted on a regional scale were carried out in urban areas with large populations and intense traffic. In addition, these sites were also heavily industrialized for economic reasons related to the distance from work to home. The results of studies showed that the incidence of CVDs was higher in low- or middle-income countries (LMIC) and developing countries [106]. The increase in industrial activities, the use of fossil fuels, the use of old and obsolete technologies in the production process, and the lack of growth in mechanization have led to a significant increase in the amount of pollutants produced by these countries. In addition, the use of manpower in heavily polluted industrial environments instead of using industrial machines, the growth of marginalization and residence in industrial areas have increased levels of exposure to high concentrations of pollutants, being an important risk factor considered to cause CVDs [107].

Therefore, the explanation for this contradiction in results can be population growth, aging, and suffering from chronic diseases, such as kidney dysfunction, as well as the additive effect of several risk factors, such as high systolic blood pressure, high blood sugar, low physical activity, high body mass index (BMI), and alcohol consumption [70]. Furthermore, limited access to clinical care and a lack of advanced diagnostic methods in low- and middle-income countries led to misdiagnosis of some CVDs [108], negatively affecting patient registries and statistics published by their health systems. Most of the articles published on this topic investigated in developed countries, while only a few papers came from developing countries. Finally, climate variability, air humidity, green space per capita, as well as the rate of industrial growth and the development of the studied society were among the factors that affected air pollution levels in different countries [109], becoming an important factor in the development of CVDs.

Based on the results presented in Table 3, the prevalence of CVDs attributed to air pollution has been investigated in a wide range of age groups. Researchers believe that the elderly are more susceptible to CVDs than other age groups due to physiological changes, smoking, sedentary lifestyle, and chronic exposure to air pollutants [110, 111]. Studies revealed that CVD frequency increases significantly after the 60 years of age, so the factor includes at least 40% of deaths in this age group [112].

Regarding the proposed mechanisms that implicate the association of air pollution with the occurrence of CVDs, air pollution was found to alter cardiovascular physiology, including heart rate and blood pressure [113], leading to an increased risk of IHD and stroke [61]. Air pollutants, specifically PM_2.5_, can enter the bloodstream after inhalation, causing systemic inflammation in the lungs and other organs [114, 115]. Furthermore, inhaled pollutants can activate lung sensory receptors, leading to an imbalance in the autonomic nervous system and increased catecholamine secretion [114, 115]. These changes can also trigger thrombosis, atherosclerosis, endothelial dysfunction, vasoconstriction, and elevated blood pressure [116, 117].

### Hospitalization due to CVDs attributed to air pollution

The results of the present systematic review showed that CVDs were one of the three main factors that led to hospital admissions as a result of exposure to air pollution. To date, numerous studies have explored the correlation between exposure to different levels of air pollutants and hospitalization [53–55]. In a time-series analysis of Xie et al. [48] investigated the relationship between short-term exposure to particulate matter (PM) and hospitalization costs of specific CVDs in China. The study concluded that exposure to PM_2.5_ could significantly increase hospital admissions and total costs of lower respiratory infections (LRI), coronary heart disease (CHD), and stroke.

The results of a national study in the USA showed that long-term exposure to low levels of PM_2.5_ (8.7 μg/m^3^) can cause hospitalization of 208,113 patients with CVDs [53]. Also, Castillo et al. [49] in a case study estimated intra-urban inequalities of exposure to this pollutant using mathematical models and datasets derived from North American satellites. The results obtained by them showed that inhalation exposure to levels of 10–17.1 μg/m^3^ PM_2.5_ caused hospitalization of 840 patients with IHD and 89 patients with stroke [49]. The results of these studies were consistent with the findings reported in China [46, 48] and Taiwan [47].

From the available evidence, it seems that PM was related to changes in hemodynamics and body homeostasis [118]. Exposure to PM was related to a decrease in heart rate variability and an increase in ventricular fibrillation, as well as higher plasma viscosity and heart rate acceleration, and even with myocardial infarction [119–121]. These effects may be clinically meaningful in patients with cardioverter defibrillators [122].

Studies have shown that exposure to inhalable pollutants can lead to increased hospital admissions and stays in the intensive care unit [52, 123, 124]. In a study conducted by Pothirat et al. [44], they examined the acute impact of air pollution on daily hospitalizations and mortality rates related to respiratory diseases and cardiovascular complications in Thailand. Their results suggested that various pollutants could contribute to various types of cardiovascular complications in patients. Specifically, the study revealed a correlation between O_3_ content and emergency hospital visits due to HF, NO_2_ content and hospital admissions due to myocardial infarction, and SO_2_ content and hospitalizations due to cerebrovascular accidents (CVA) [44]. Another recent study showed that an increase of 10 μg/m^3^ NO_2_ resulted in a risk increase of 1.9% (RR: 1.019, 95% CI: 1.005 to 1.032) for hospital admissions for CVDs at lag 0–2 days. Specifically, the risk increased by 2.1% (1.021, 1.006 to 1.036) for hospitalization due to IHD, and by 2.1% (1.021, 1.006 to 1.035) for hospitalization due to ischemic stroke [55].

However, this study did not find any significant relationship between NO_2_ and hospital admissions due to arrhythmias, HF, and hemorrhagic stroke [55]. Differences in the results of other studies might be due to the number of subjects, industrial development, and socioeconomic levels of the investigated populations.

### Disability due to CVDs attributed to air pollution

The results of the studies included in this systematic review indicate that PM_2.5_ causes a two-fold increase in DALYs associated with CVDs. The 2015 GBD study identified PM_2.5_ as the cause of 4.2 million deaths and 103.1 million DALYs worldwide [9], which is consistent with the results of the study by Sang et al. [73]. Research carried out in 204 countries during the 1990–2019 period estimated that exposure to PM_2.5_ led to a two-fold increase in DALYs related to stroke and IHD, with IHD, stroke, and COPD being the three main causes of death, and DALYs attributed to this pollutant [73]. Furthermore, the European Environment Agency (EEA) reported 63,100 deaths and 710,900 years of YLL attributed to PM_2.5_ in Germany in 2018 [125]. Meanwhile, Lelieveld et al. [67] investigated the burden of CVDs attributed to PM_2.5_ in 28 European countries and revealed 14 million YLL, which is 19.7 times more than reported in EEA statistics.

The contribution of non-renewable energy sources to PM_2.5_ emission and pollution, especially in urban areas, is undeniable. According to the Lancet report (2023), Asia accounted for 77% of all deaths attributed to fuel-related particulate matter, with 1.3 million deaths. Asia, where 43% of its total energy is coal-fired, has the highest mortality rate from coal-derived PM_2.5_ among other continents (11 deaths per 100,000 people) [12]. Europe, by adopting air quality control measures, saw a 5.2% reduction in the share of coal-derived energy during 2005, reducing mortality rates related to ambient PM_2.5_ by 36%, 44% of this is a result of the reduction of pollution attributed to coal. However, Europe in 2020 had the highest death rates from outdoor PM_2.5_ pollution (69 deaths per 100,000 people) and dirty energy sources, such as biomass and fossil fuels (38 deaths per 100,000 people) [12].

So far, many studies on a national and international scale have shown the increase in disability cases associated with CVDs in recent years. Feigin et al. [70] in their systematic analysis on the global, regional, and national burden of stroke during the years 1990–2019 revealed that exposure to levels higher than 8.8 μg/m^3^ PM_2.5_ caused 28.7 million DALYs worldwide [70], which was consistent with the results obtained by Sang et al. [73]. When examining the global burden of disease attributable to ambient PM_2.5_ in 204 countries, Sang et al. [73] concluded that the DALY index for stroke increased from 18 million in 1990 to 35 million in 2019 (approximately a two-fold increase) [73], but on the other hand, some studies have produced contradictory results.

The study by Campos Caldeira Brant et al. [59] found that the DALY rate associated with exposure to PM_2.5_ for Brazilian residents in 2019 was 336 years, reflecting a 75% decrease compared to the DALY rate in 1990. Similar results were found in the study of Rueda et al. [85] on the burden of diseases caused by PM_2.5_ in the Kingdom of Saudi Arabia (KSA) [85], which showed that DALY and YLL caused by IHD increased by approximately 3 and 1.2 times, respectively, during the years 1990–2010 and 2010–2017. The YLD of IHD also increased markedly by 3294.35 times from the value of 201 in 1990 to the value of 662,167 in 2010 [85]. The extensive use of fossil fuels, the development of industry and refineries, and proximity to the Great Arabian desert, which is the main source of natural PM [126], resulted in the increased disability caused by CVDs as the consequence of exposure to PM.

However, significant technological advances and the implementation of global corrective measures were able to have a positive impact on improving the health of communities in these countries by increasing the employment rate in the clean energy sector, as well as green lending by the World Bank and regional development banks. Furthermore, the significant increase in investment in the renewable energy sector in recent years has led to an important step towards achieving a reduction in fossil fuel consumption. The increase in the investment rate in 2022 was 15% compared to 2021 and 51% compared to 2015. Reduction in the usage of non-renewable fuel sources caused the decrease in exposure to air pollutants and related adverse health effects [127–129].

The occurrence and development of CVDs is a complex health issue influenced by several factors, including difficult to control variables, such as traffic noise, daily stress, lifestyle, and regional customs [130, 131]. According to what was said, although the increase in PM_2.5_ levels has been associated with an increase in cases of disability caused by exposure to this pollutant, the reason for the decrease in the DALY rate reported in the Brazilian [59] and Saudi Arabia [85] studies can be attributed to the aforementioned factors.

### Mortality due to CVDs attributed to air pollution

Investigation of the included studies showed that exposure to different levels of air pollutants, especially PMs, has a direct relationship with the increase in CVDs mortality. The findings of this systematic review are consistent with the results published by the WHO in 2016, which reported that 74% of global deaths (2,161,550 cases) attributed to air pollution were related to CVDs, particularly stroke and IHD [132]. In the study on the global burden of CVDs in India, air pollution was identified as the main cause of approximately one third of CVDs incidences, namely 31.1% (UI 29.0–33.4) during the years 1990–2016, resulting in a total mortality rate of 28.1% (95% UI 26.5–29.1) [7]. Furthermore, the study by Lelieveld et al. [67] showed that ambient air pollution in Europe was responsible for approximately 790,000 deaths per year (95% confidence interval [95% CI] 645,000–934,000), of which 40–80% occurred due to cardiovascular events. Furthermore, eliminating greenhouse gas emissions from fossil fuels could reduce annual death rates in Europe by 434,000 (95% CI 355,000–509,000) cases [67].

Anthropogenic activities cause emissions of man-made greenhouse gases (GHGs) such as hydrofluorocarbons (HFCs), perfluorocarbons (PFCs), and sulphur hexafluoride (SF_6_), as well as increases in natural GHGs such as carbon dioxide (CO_2_), nitrous oxide (N_2_O), methane (CH_4_), and water vapour. The rate and the amount of GHGs emissions in recent decades has led to the global issue of climate change and the implementation of various measures to mitigate this environmental problem. One of the most important global actions is the Paris Agreement [11], which was ratified in 2015 at the United Nations Climate Change Conference (COP21) in Paris, France by 196 countries, representing 95% of the countries responsible for anthropogenic greenhouse gas emissions. The Paris Agreement priority goal is to keep the average global temperature increase below 2 ºC above preindustrial levels (the preferable limit of 1.5 ºC). This can be achieved only by significant reductions in all GHGs emissions. The success in achieving this objective depends on the reduction of industrial activities with high pollutant emissions, the use of Best Available Technologies (BATs) in the production of vehicles to reduce pollutant emissions, the encouragement of the production and usage of electric vehicles, the use of clean fuels instead of fossil ones, in combating deforestation and increasing the forest cover. The latter is considered to be a very effective solution in reducing air pollution and the related burden of diseases [11]. Furthermore, the analyzed studies indicated that the reduction in air pollution is estimated to prevent many of the current 3.3 million deaths resulting from exposure to anthropogenic PM_2.5_ [12].

For example, the national cohort study from the USA with a six-year follow-up revealed that the increase of 1 μg/m^3^ in the mean annual concentration of PM_2.5_ was associated with an increase in the rate of cardiovascular events (hazard ratio HR, 1.02 [95% CI, 1.01–1.02]) and specific mortality (HR, 1.02 [95% CI, 1.02–1.03]) of associated CVDs [53]. These results suggest that chronic exposure to particulate matter, even at relatively low levels, has a potential positive association with CVDs and mortality, especially for chronic diseases.

Toxicological studies revealed that PM_10_ and PM_2.5_ can cause lung inflammation, oxidative stress, and cytotoxicity, leading to cardiovascular damage and even death [133, 134]. Some researchers argue that exposure to PM_2.5_ causes higher cytotoxicity than exposure to PM_10_ [135]. Previous studies also reported a significant relationship between exposure to O_3_ and cardiovascular morbidity and mortality [62, 78, 82]. Yin et al. [34] investigated 272 cities in southern China and found that the increase by 10 µg/m^3^ in the maximum 8-h O_3_ concentration led to a 0.66% (95% CI: 0.02%, 1.30%) increase in daily mortality due to hypertension in the general local population. Similar results were presented in the study by Li et al. [84] on the short-term effects of exposure to environmental NO_2_.

### Costs due to CVDs attributed to air pollution

The increase in morbidity, disability, and death caused by CVDs attributed to air pollution imposes huge costs on governments involved in this environmental dilemma. Several studies have investigated the economic losses associated with chronic exposure to ambient air pollution [136, 137], but only a few examined the related economic burden (Table 5) [46, 48, 52, 56]. The results of the surveys showed that exposure to different levels of pollutants could increase health costs, reduce labor supply, and cause job losses. Short-term exposure to air pollutants was found to increase hospital admissions due to cardiorespiratory diseases, causing the government to significantly increase the costs spent on public health [123, 138, 139]. According to Xie et al. [48], the estimated costs associated with the most common CVDs related to short-term exposure to PM_2.5_ (49.7 μg/m^3^) were 220 million CNY (US$ 30.4 million) for LRI, 458 million CNY (US$ 63.2 million) for CHD, and 410 million CNY (US$ 56.6 million) for stroke. These numbers represented 1.45–2.05% of all hospital admission costs [48]. Workday loss related to CVDs due to exposure to air pollution calculated by Yao et al. [56] revealed that NO_2_ with a concentration of 30.23 μg/m^3^ caused the highest economic burden (604.02 billion CNY or US$ 83.04 billion), while SO_2_ with a level of 18.14 μg/m^3^caused the lowest (195.28 billion CNY or US$ 27.9 billion). Yip et al. [140] revealed in their studies a four-fold increase in government health expenditures for health care from 2008 to 2017, which is consistent with the study of Dobkin et al. [141]. Hospital admissions can significantly increase out-of-pocket medical expenses, unpaid medical bills, reduced income, and even bankruptcy [141]. Direct costs will be much higher considering also outpatient visits.

From the available evidence, it appears that air pollution plays a very significant role in increasing the economic costs of the health system. The monetary costs of premature deaths attributed to air pollution in 2020 were estimated at 2.2 trillion US dollars, which was equivalent to 2.4% of the gross world product [12].

Although many efforts have been made to solve this global environmental issue, attempts to maintain people’s health and safety have so far been insufficient and unfair [142]. Obviously, some actions during recent years also played an important role in neutralizing the corrective measures. The demand for economic recovery after the COVID-19 pandemic crisis, the war outbreak in Ukraine in 2021, the subsequent imposition of economic sanctions and the disruption of oil and gas supplies, and extreme weather events after the El Niño phenomenon in 2023, has affected energy production and caused dramatic price increases. Unfortunately, it also caused the return to fossil fuels in many anthropogenic activities and new sources of oil and gas prospecting [143, 144]. The increase in energy prices caused significant profits for oil and gas companies ($4 trillion in 2022 versus an average of $1.5 trillion in the previous years), resulting in a further decrease in the company’s adherence to the implementation of the Paris Agreement [145–147].

Furthermore, the gradual elimination of fossil fuels and the transition to clean and renewable energy have become a significant challenge due to several reasons. Among these reasons are a 10% increase in global investment in fossil fuels in 2022, direct net subsidies provided by governments, and an increase in bank lending to the fossil fuel sector by the top 55% of private banks [148, 149]. These conditions were associated with imposing a high economic and health burden and a high death rate attributed to air pollution, especially for local populations, making countries with abundant natural renewable energy resources, such as Africa, Asia, and South and Central America, lag in the transition to clean energy. Therefore, it is crucial to achieve equality in access to clean fuel technologies, to support sustainable development, to reduce global inequalities, and as a result, to achieve global health goals.

Providing green energy transfer subsidies and increasing lending to the renewable energy sector are required and undertaken to reduce air pollution and greenhouse gases in low-HDI countries. Among these, the following efforts are vitally important to achieve the goals of reducing air pollutants and also reducing the costs imposed on the health system: Efforts to 1) improve sustainable city design and spatial management focusing on health issues; 2) reduce pollutant emissions from buildings, and increase the flexibility of communities in the face of climate risks; and 3) encourage governments to develop electric public transportation and impose strict tax laws for companies in case of violation of emission laws.

In addition, it can be very useful to take advantage of artificial intelligence-based long-term estimators and policymakers that have recently been developed to address challenging health problems [150]. The use of this tool can help to estimate future losses, determine and prioritize effective interventions and determine the most optimal conditions for applying interventions.

Our study revealed multiple strengths. First, to our knowledge, it was the first systematic review exploring the global burden of CVDs related to air pollution. Second, we conducted a systematic search without restrictions on publication date, study type, or countries under review, except for the language of the studies (only English). This approach allowed us to examine more studies, to analyze ample data, and to conclude on the air pollution in the burden of CVDs worldwide. However, since we did not have access to the full text of certain studies that met the eligibility criteria (7 studies), we are aware that it could affect the global picture of the conclusions presented in this systematic review.

## Conclusion

Ambient air pollutants, especially PM_2.5_, are known to trigger the occurrence of CVDs. Hypertension was revealed to have the highest prevalence, while coronary heart disease was documented to have the lowest prevalence among other types of CVDs caused by air pollution. Based on the reviewed studies, CVDs were shown to be one of the three main factors that lead to hospital admissions as a result of exposure to air pollution. Furthermore, disabilities such as DALY, YLD, and YLL caused by CVDs, particularly stroke and IHD, increased significantly as a consequence of the ambition of the countries to improve the degree of industrialization. Thus, related air pollution is higher for obvious reasons in low- and middle-income and developing countries. Moreover, the consequence is not only environmental pollution itself, but also the significant number of CVD cases and deaths in the global population. In terms of economic burden, there was a lack of comprehensive research on the economic impact of CVDs due to air pollution. This indicates either an underestimation of the impact of this risk factor or a gap in research efforts. Although it is evident that CVDs linked to air pollutants impose a substantial constraint on public health, delve into this aspect could potentially offer a strategic vantage point for mitigating the burden of CVDs.

### Supplementary Information

**Supplementary Material 1.**

**Supplementary Material 2.**

## Data Availability

The datasets used and/or analyzed during the current study are available from the corresponding author on reasonable request.
