# Supplementary material for: Burden of cardiovascular disease attributed to air pollution: a systematic review
Source: Global Health. 2024 May 3;20:37. doi: 10.1186/s12992-024-01040-0 (PMC11069222; doi:10.1186/s12992-024-01040-0)
Supplement: Supplementary file 1 — Supplementary Material 1. [file 12992_2024_1040_MOESM1_ESM.docx]

**Table A1**. Disability due to cardiovascular diseases (CVDs) attributed to air pollution as reported in the analyzed studies

| **Author (year)** | **Title** | **Country (city)** | **Number of samples** | **Gender** | **Age (years)** | **Pollutant** | **Mean concentration [μg/m³]** | **Disability-adjusted life years (DALY)** | **Years lost due to disability  (YLD)** | **Years of life lost (YLL)** |
| --- | --- | --- | --- | --- | --- | --- | --- | --- | --- | --- |
| Myriam Tobollik (2015) | Burden of Outdoor Air Pollution in Kerala, India—A First Health Risk Assessment at State Level | India  (Kochi, Kozhikode, Thrissur, Malappuram, Trivandrum, Kollam) | 81,636 | Male-female | <1 - ≥70 | PM_10_ | 31-67 | Nd | Nd | CVD: 47,966 |
| Valery L Feigin (2016) | Global burden of stroke and risk factors in 188 countries, during 1990–2013: a systematic analysis for the Global Burden of Disease Study 2013 | Global  (188 countries) | Nd | Male-female | 15 - ≥70 | PM_2.5_ | Nd | Stroke: 19,047,618 | Nd | Nd |
| Aaron J Cohen (2017) | Estimates and 25-year trends of the global burden of disease attributable to ambient air pollution: an analysis of data from the Global Burden of Diseases Study 2015 | Global | 4,241,100 | Male-female | <5 - >70 | PM_2.5_, O_3_ | PM_2.5_ = 39.7-44.2  O_3_ = 106.86-112.88 | **In 1000 individuals:**  IHD: 32,406.0  CEV: 19,242.8 | Nd | Nd |
| Tunde O Etchie (2017) | The health burden and economic costs averted by ambient PM_2.5_ pollution reductions in Nagpur, India | India  (Nagpur) | 3,300 | Male-female | <5 - ≥25 | PM_2.5_ | 34 | IHD: 47,000  Stroke: 21,000 | Nd | Nd |
| Jing Huang (2018) | The burden of ischemic heart disease related to ambient air pollution exposure in a coastal city in South China | China  (Ningbo) | 10,322 | Male-female | <75 - ≥75 | PM_2.5_, SO_2_, NO_2_ | PM_2.5_ = 49.58  SO_2_ = 21.34  NO_2_ = 43.41 | Nd | Nd | IHD: 184,064.8 |
| Dorairaj Prabhakaran (2018) | The changing patterns of cardiovascular diseases and their risk factors in the states of India: the Global Burden of Disease Study 1990–2016 | India  (national) | 1,316 million | Male-female | ≥10 | PM_2.5_ | Nd | CVD: 4.38%  IHD: 3.13%  Stroke: 1.15% | Nd | Nd |
| Xiao Lin (2018) | The burden of cardio-cerebrovascular disease and lung cancer attributable to PM_2.5_ for 2009, Guangzhou: a retrospective population-based study | China  (Guangzhou) | 4,700,000 | Male-female | 37 | PM_2.5_ | 54.1 | Nd | Nd | **Per year per 100,000 individuals:**  CVD: 317.1 |
| Gerardo Sanchez Martinez (2018) | Health Impacts and Economic Costs of Air Pollution in the Metropolitan Area of Skopje | Republic of Macedonia  (Skopje) | 53,1524 | Male-female | ≥30 | PM_2.5_ | 49.2 | Nd | Nd | CVD: 1,813,000 |
| Zhan Wang (2019) | Impact of air pollution waves on the burden of stroke in a megacity in China | China  (Tianjin) | 11.0 million | Male-female | Nd | PM_10_, SO_2_, NO_2_ | PM_10_ = 98.4  SO_2_ = 57.3  NO_2_ = 43.2 | Nd | Nd | Stroke: 1,026,505 |
| Mansour Shamsipour (2019) | National and sub-national exposure to ambient fine particulate matter (PM_2.5_) and its attributable burden of disease in Iran from 1990 to 2016 | Iran  (National) | 41,272 | Male-female | Nd | PM_2.5_ | 21.7 - 35.4 | Nd | Nd | IHD: 2,271,078  Stroke: 514,650 |
| Jos Lelieveld (2019) | Cardiovascular disease burden fromambient air pollution in Europe reassessed using novel hazard ratio functions | Europe  (28 countries) | 2,138,000 | Male-female | 65 - 70 | PM_2.5_ | Nd | Nd | Nd | CVD: 14,000,000 |
| Jing Huang (2020) | How Birth Season A ects Vulnerability to the E ect of Ambient Ozone Exposure on the Disease Burden of Hypertension in the Elderly Population in a Coastal City in South China | China  (Ningbo) | 5.83 million | Male-female | ≥75 | O_3_ | 93.3 | Nd | Nd | Hypertension: 70,014 |
| Yanfeng Jiang (2020) | Stroke burden and mortality attributable to ambient fine particulate matter pollution in 195 countries and territories and trend analysis from 1990 to 2017 | Global  (195 countries) | 104.2 million | Male-female | 35 - ≥80 | PM_2.5_ | Nd | 10.5 million | Nd | Nd |
| Jie Li (2020) | Ambient ozone pollution and years of life lost: Association, effect modification, and additional life gain from a nationwide analysis in China | China  (National) | 403 million | Male-female | <65 - ≥65 | O_3_ | 86.9 | Nd | Nd | **Average daily mortality:**  CVD: 637 |
| Sanju Bhattarai (2020) | Cardiovascular disease trends in Nepal – An analysis of global burden of disease data 2017 | Nepal  (National) | Nd | Male-female | 1 - ≥80 | PM_2.5_ | Nd | **Per 100,000** **population**  IHD ≈ 350  Stroke ≈ 420 | Nd | Nd |
| Peng Yin (2020) | The effect of air pollution on deaths, disease burden, and life expectancy across China and its provinces, 1990–2017: an analysis for the Global Burden of Disease Study 2017 | China  (national) | 1.24 million | Male-female | <5 - >70 | PM_2.5_, O_3_ | PM_2.5_= 52.7  O_3_= 128.31 | **Per 1000 individuals**  IHD: 4,506.4  Stroke: 4,290.9 | Nd | Nd |
| Wenyuan Yu (2020) | Burden of ischemic heart disease and stroke attributable to exposure to atmospheric PM_2.5_ in Hubei province, China | China  (Hubei) | 57.24 million | Male-female | 30 - 69 | PM_2.5_ | 54.50 | **Per 1000 individuals**  IHD: 313.39  Stroke: 791.90 | Nd | Nd |
| Dieyi Chen (2020) | Utilizing daily excessive concentration hours to estimate cardiovascular mortality and years of life lost attributable to fine particulate matter in Tehran, Iran | Iran  (Tehran) | 106,180 | Male-female | 0 - ≥75 | PM_2.5_ | 34.7 | Nd | Nd | **Daily-person years:**  CVD: 951.3  Stroke: 167.8  IHD: 264.4  SCD: 276.2 |
| Seyed M. Karimi (2020) | Continuous Exposure to Ambient Air Pollution and Chronic Diseases: Prevalence, Burden, and Economic Costs | Iran  (Tehran) | 67,049 | Male-female | 49.1 - 68.6 | CO, NO_2_, O_3_, PM_10_ | CO = 4,124.17  NO_2_ = 101.6  O_3_ = 42.9  PM_10_ = 101.7 | Nd | Nd | **Per 100,000 individuals:**  Stroke: 1,692-5,259  Hypertension: 4,823-14,306 |
| Lina Wang (2021) | Global burden of ischemic heart disease attributable to ambient PM_2.5_ pollution from 1990 to 2017 | Global  (195 countries) | Nd | Male-female | Nd | PM_2.5_ | Nd | IHD: 12,381,000 – 21,928,000 | Nd | Nd |
| Valery L Feigin (2021) | Global, regional, and national burden of stroke and its risk factors, 1990–2019: a systematic analysis for the Global Burden of Disease Study 2019 | Global  (204 countries) | 12.22 million | Male-female | ≤70 - >70 | PM_2.5_ | > 8.8 | Stroke: 28.7 million | Nd | Nd |
| Qingfeng Ma (2021) | Temporal trend and attributable risk factors of stroke burden in China, 1990–2019: an analysis for the Global Burden of Disease Study 2019 | China  (national) | 3.94 million | Male-female | 15 - ≥80 | PM_2.5_ | Nd | **Per 1000 individuals:**  Stroke: 12,847  Ischemic stroke: 5,741  Intracerebral hemorrhage: 6,406  Subarachnoid hemorrhage: 706 | Nd | Nd |
| Jie Li (2021) | The association between ozone and years of life lost from stroke, 2013-2017: a retrospective regression analysis in 48 major Chinese cities | China  (48 cities) | 2.15 million | Male-female | <65 - ≥65 | O_3_ | 86.9 | Nd | Nd | Stroke: 26.3 million |
| Yu Wang (2021) | The impact of carbon monoxide on years of life lost and modified effect by individual- and city-level characteristics: Evidence from a nationwide time-series study in China | China  (48 cities) | Nd | Male-female | <65 - ≥ 65 | CO | 1.2 | Nd | Nd | CVD: 636.6  CHD: 236.0  Stroke: 300.1 |
| Jie Li (2021) | Short-term effects of ambient nitrogen dioxide on years of life lost in 48 major Chinese cities, 2013-2017 | China  (48 cities) | 403 million | Male-female | 0 - ≥75 | NO_2_ | 39.7 | Nd | Nd | **Average daily mortality:**  Stroke: 637 |
| D. Rojas-Rueda (2021) | Ambient particulate matter burden of disease in the Kingdom of Saudi Arabia | Kingdom of Saudi Arabia  (national) | 19,569 | Male-female | 1 - ≥95 | PM_2.5_ | 87.9 | IHD:  1990: 34,654  2010: 109,167  2017: 138,041  Stroke:  1990: 14,017  2010: 28,973  2017: 34,334 | IHD:  1990: 201  2010: 662,167  2017: 992  Stroke:  1990: 1,099  2010: 3,099  2017: 4,537 | IHD:  1990: 34,654  2010: 108,505  2017: 137,049  Stroke:  1990: 12,918  2010: 25,874  2017: 29,797 |
| Guijie Luan (2021) | Associations between ambient air pollution and years of life lost in Beijing | China  (national) | 386,695 | Male-female | ≤65 - >65 | Nd | API = 84 | Nd | Nd | CVD: 994  IHD: 428  Stroke: 456 |
| Alen Juginović (2021) | Health impacts of air pollution exposure from 1990 to 2019 in 43 European countries | Europe  (43 countries) | Nd | Male-female | <5 - >70 | PM_2.5_ | 20.8 - 13.8 | IHD: 124.8  Stroke: 64.1 | Nd | **Per 100,000 individuals:**  IHD: 60-850  Stroke: 75-1650 |
| Shaowei Sang (2022) | The global burden of disease attributable to ambient fine particulate matter in 204 countries and territories, 1990–2019: A systematic analysis of the Global Burden of Disease Study 2019 | Global  (204 countries) | 6,000,000 | Male-female | 0 – ≥95 | PM_2.5_ | Nd | IHD:  1990: 1× 10^7^  2019: 2× 10^7^  Stroke:  1990: 1.8× 10^7^  2019: 3.5× 10^7^ | Nd | Nd |
| Benjamim M. Varieur (2022) | Air Pollution, Political Corruption, and Cardiovascular Disease in the Former Soviet Republics | Soviet republics  (Eastern Europe-Western Europe) | 178,000 | Male-female | Nd | PM_2.5_ | 12.4 - 20.0 | CVD: 4,010,000 | Nd | Nd |
| Luisa Campos Caldeira Brant (2022) | Burden of Cardiovascular diseases attributable to risk factors in Brazil: data from the "Global Burden of Disease 2019" study | Brazil  (national) | Nd | Male-female | Nd | PM_2.5_ | 2.4 - 5.9 | **CVD**  **Per 100,000**  **inhabitants:**  1990: 1,334  2019: 336 | Nd | Nd |
| Myriam Tobollik (2022) | Burden of Disease Due to Ambient Particulate Matter in Germany—Explaining the Differences in the Available Estimates | Germany  (national) | 15,600 | Male-female | <1 - ≥95 | PM_2.5_ | 13.7 - 10.8 | IHD: 85,483  Stroke: 38,417 | IHD: 16,293  Stroke: 13,399 | IHD: 85,483  Stroke: 25,019 |
| Hugo Grisales-Romero (2023) | Local attributable burden disease to PM_2.5_ ambient air pollution in Medellín, Colombia, 2010–2016 | Colombia  (Medellín) | 3,873 | Male-female | 0 - ≥80 | PM_2.5_ | 35.8 | IHD: 30,748  CEV: 6,197 | IHD: 2,031  CEV: 1,413 | IHD: 28,717  CEV: 4,784 |
|  | Nd – Not defined  IHD – Ischemic Heart Disease  CVD – Cardiovascular Disease  CHD – Coronary Heart Disease  CEV – Cerebrovascular Disease  AMI – Acute Myocardial Infarction  HHD – Hypertensive Heart Disease  SCD – Sudden Cardiac Death  API – Air Pollution Index | | | | | | | | | |
