# Supplementary material for: Burden of cardiovascular disease attributed to air pollution: a systematic review
Source: Global Health. 2024 May 3;20:37. doi: 10.1186/s12992-024-01040-0 (PMC11069222; doi:10.1186/s12992-024-01040-0)
Supplement: Supplementary file 2 — Supplementary Material 2. [file 12992_2024_1040_MOESM2_ESM.docx]

**Table A2**. Mortality due to cardiovascular diseases (CVDs) attributed to air pollution as reported in the analyzed studies

| **Author (year)** | **Title** | **Country (city)** | **Number of samples** | **Gender** | **Age (years)** | **Pollutant** | **Mean concentration [μg/m^3^]** | **Mortality rate** | **Number of deaths** |
| --- | --- | --- | --- | --- | --- | --- | --- | --- | --- |
| Myriam Tobollik (2015) | Burden of Outdoor Air Pollution in Kerala, India - A First Health Risk Assessment at State Level | India (Kochi, Kozhikode, Thrissur, Mallapuram, Trivandrum, Kollam) | 81,636 | Male-female | <1 - ≥70 | PM_10_ | 31 - 67 | **Per 100,000 individuals:**  CVD: 333 | CVD: 26,296 |
| Rakesh Ghosh (2016) | Near-Roadway Air Pollution and Coronary Heart Disease: Burden of Disease and Potential Impact of a Greenhouse Gas Reduction Strategy in Southern California | USA (California) | 1.2 million | Male-female | 45 - ≥85 | PM_2.5_, Elemental carbon (EC) | Nd | **Per 1,000 individuals:**  CHD: 3.4 | **CHD:**  EC: 690  PM_2.5_: 1,900 |
| Chayut Pinichka (2017) | Burden of disease attributed to ambient air pollution in Thailand: A GIS-based approach | Thailand (national) | Nd | Male-female | Nd | PM_2.5_, PM_10_, NO_2_ | PM_2.5_= 27.2  PM_10_= 54.5  NO_2_= 32.17 | Nd | **CVD**  **In 1000 individuals:**  PM_2.5_: 153.6  PM_10_: 40.24 |
| Peng Yin (2017) | Long-term Fine Particulate Matter Exposure and Non accidental and Cause-specific Mortality in a Large National Cohort of Chinese Men | China (45 districts/ counties) | 189,793 | Male | 54.8 | PM_2.5_ | 43.7 | Nd | CVD: 18,859  IHD: 3,752  Stroke: 11,301 |
| Jianlin Hu (2017) | Premature Mortality Attributable to Particulate Matter in China: Source Contributions and Responses to Reductions | China (national) | 1.30 million | Male-female | ≥30 | PM_2.5_ | 62.6 | Nd | CEV: 742,000  IHD: 306,000 |
| Aaron J Cohen (2017) | Estimates and 25-year trends of the global burden of disease attributable to ambient air pollution: an analysis of data from the Global Burden of Diseases Study 2015 | Global | 4,241,100 | Male-female | <5 - >70 | PM_2.5_, O_3_ | PM_2.5_ = 39.7 - 44.2  O_3_ = 56.8 - 60.9 | **Per 100,000 individuals:**  IHD: 23.6  CEV: 14 | IHD:1521.1  CEV: 898.1 |
| Scott Weichenthal (2017) | Impact of Oxidant Gases on the Relationship between Outdoor Fine Particulate Air Pollution and Nonaccidental, Cardiovascular, and Respiratory Mortality | Canada (national) | 2,448,500 | Male-female | 25 - 89 | PM_2.5_ | 7.37 | Nd | CVD: 77,000 |
| Tunde O. Etchie (2017) | The health burden and economic costs averted by ambient PM_2.5_ pollution reductions in Nagpur, India | India (Nagpur) | 3,300 | Male-female | <5 - ≥25 | PM_2.5_ | 34 | Nd | IHD: 1,800  Stroke: 900 |
| Hualiang Lin (2018) | Daily exceedance concentration hours: A novel indicator to measure acute cardiovascular effects of PM_2.5_ in six Chinese subtropical cities | China (Guangzhou, Shenzhen, Zhuhai, Foshan, Dongguan, and Jiangmen) | 49.1 million | Male-female | Nd | PM_2.5_ | 42.23 | Nd | **Average daily mortality:**  CVD: 25  IHD: 9  AMI: 5  CEV: 8 |
| Xiaojie Wang (2018) | Ambient coarse particulate pollution and mortality in three Chinese cities: Association and attributable mortality burden | China (Guangzhou, Foshan, and Jiangmen) | 24.1 million | Male-female | Nd | Coarse particles pollution (PMc) | 20.54 - 23.44 | Nd | **Average daily mortality:**  CVD: 39 |
| Xiao Lin (2018) | The burden of cardio-cerebrovascular disease and lung cancer attributable to PM_2.5_ for 2009, Guangzhou: a retrospective population-based study | China (Guangzhou) | 4,700,000 | Male-female | 37 | PM_2.5_ | 54.1 | **Per 100,000 individuals:** CVD: 155.5 | CVD: 3,923 |
| Chen Chen (2018) | Short-term exposures to PM_2.5_ and cause-specific mortality of cardiovascular health in China | China (30 counties) | 23.7 million | Male-female | <65, 65 - 74 - >74 | PM_2.5_ | 71.67 | Nd | CVD: 164,061  CHD: 69,041  AMI: 26,098  Stroke: 49,669 |
| Gerardo Sanchez Martinez (2018) | Health Impacts and Economic Costs of Air Pollution in the Metropolitan Area of Skopje | Republic of Macedonia (Skopje) | 531,524 | Male-female | ≥30 | PM_2.5_ | 49.2 | Nd | CVD: 179,900 |
| Jong-Hun Kim (2018) | Premature Deaths Attributable to Long-term Exposure to Ambient Fine Particulate Matter in the Republic of Korea | Korea (national) | 17,203 | Male-female | ≥65 | PM_2.5_ | 30.2 | Nd | IHD: 3,432  Ischemic stroke: 5,382  Hemorrhagic stroke: 3,452 |
| Zhan Wang (2019) | Impact of air pollution waves on the burden of stroke in a megacity in China | China (Tianjin) | 11.0 million | Male-female | Nd | PM_10_, SO_2_, NO_2_ | PM_10_ = 98.4  SO_2_ = 57.3  NO_2_ = 43.2 | Nd | Stroke: 81,600 |
| Lizhong Xu (2019) | Spatial disequilibrium of fine particulate matter and corresponding health burden in China | China (336 cities) | Nd | Male-female | Nd | PM_2.5_ | 45 | **Per 10,000 individuals**:  Stroke: 18.8  IHD: 15.6  HHD: 3.8 | Stroke: 0.52 million  IHD: 0.43 million  HHD: 0.10 million |
| Lan Yao (2019) | Contribution of transregional transport to particle pollution and health effects in Shanghai during 2013–2017 | China (Shanghai) | 54,674 | Male-female | Nd | PM_2.5_, PM_10_ | PM_2.5_:  2013= 62  2014= 52  2015= 53  2016= 45  2017= 39  PM_10_:  2013= 82  2014= 71  2015= 68  2016= 59  2017= 57 | CVD:  2013: 1.96%  2014: 2.00%  2015: 1.99%  2016: 2.01%  2017: 2.14% | CVD:  2013: 652  2014: 548  2015: 572  2016: 428  2017: 455 |
| Mansour Shamsipour (2019) | National and sub-national exposure to ambient fine particulate matter (PM_2.5_) and its attributable burden of disease in Iran from 1990 to 2016 | Iran (national) | 41,272 | Male-female | Nd | PM_2.5_ | 21.7 - 35.4 | **Per 100,000 individuals:**  IHD: 31,363  Stroke: 7,012 | IHD: 38.4  Stroke: 8.5 |
| Alireza Khajavi (2019) | Impact of temperature and air pollution on cardiovascular disease and death in Iran: A 15-year follow-up of Tehran Lipid and Glucose Study | Iran (Tehran) | 9,731 | Male-female | 47.7 | CO, O_3_, PM_2.5_, PM_10_, NO_2_, SO_2_ | Nd | Nd | CVD: 725 |
| Benjamin Bowe (2019) | Burden of Cause-Specific Mortality Associated With PM_2.5_ Air Pollution in the United States | USA (national) | 4,522,160 | Male-female | 64.1 | PM_2.5_ | 4.8 - 20.1 | **Per 100,000 individuals:**  CVD: 17.3  CEV: 12.5  Hypertension: 9.5 | CVD: 56,070.1  CEV: 40,466.1  Hypertension: 30,696.9 |
| Chris C. Lim (2019) | Mediterranean Diet and the Association Between Air Pollution and Cardiovascular Disease Mortality Risk | USA (California, Florida, Louisiana, New Jersey, North Carolina, and Pennsylvania) | 548,845 | Male-female | 62.2 | PM_2.5_, NO_2_ | PM_2.5_ = 12.9  NO_2_ = 25.02 | Nd | CVD: 39,532  IHD: 22,329  CEV: 5,592  Dysrhythmias, heart failures, and cardiac arrests: 6,811 |
| Jos Lelieveld (2019) | Cardiovascular disease burden fromambient air pollution in Europe reassessed using novel hazard ratio functions | Europe (28 countries) | 2,138,000 | Male-female | 65 - 70 | PM_2.5_ | Nd | Nd | IHD: 313,000  CEV: 64000  CVD: 377,000 |
| Jing Huang (2020) | How Birth Season A ects Vulnerability to the E ect of Ambient Ozone Exposure on the Disease Burden of Hypertension in the Elderly Population in a Coastal City in South China | China (Ningbo) | 5.83 million | Male-female | ≥75 | O_3_ | 93.3 | Nd | Hypertension: 6,525 |
| Yanfeng Jiang (2020) | Stroke burden and mortality attributable to ambient fine particulate matter pollution in 195 countries and territories and trend analysis from 1990 to 2017 | Global (195 countries) | 104.2 million | Male-female | 35 - ≥80 | PM_2.5_ | Nd | Nd | Stroke: 0.4 million |
| Xueli Yang (2020) | Associations of long-term exposure to ambient PM_2.5_ with mortality in Chinese adults: A pooled analysis of cohorts in the China-PAR project | China (national) | 116,821 | Male-female | 51.6 | PM_2.5_ | 64.9 | Nd | Cardio-metabolic mortality: 2,507 |
| Jie Li (2021) | Ambient ozone pollution and years of life lost: Association, effect modification, and additional life gain from a nationwide analysis in China | China (national) | 403 million | Male-female | <65 - ≥65 | O_3_ | 86.9 | Nd | **Average daily mortality:**  CVD: 53 |
| Jun Yang (2020) | Fine particulate matter constituents and cause-specific mortality in China: A nationwide modelling study | China (national) | Nd | Male-female | 0 – ≥75 | PM_2.5_, Organic carbon (OC), Elemental carbon (EC), Sulphate (SO_4_^2-^), nitrate (NO^3-^) and ammonium (NH4^+^) | PM_2.5_= 60.6 OC=7.8  EC= 3.3  SO_4_= 14.8 NO_3_= 13.5  NH_4_ =8.9 | Nd | **Average daily mortality:**  CVD: 4  IHD: 1  Stroke: 2  MI: 1 |
| Peng Yin (2020) | The effect of air pollution on deaths, disease burden, and life expectancy across China and its provinces, 1990–2017: an analysis for the Global Burden of Disease Study 2017 | China (national) | 1.24 million | Male-female | <5 - >70 | PM_2.5_, O_3_ | PM_2.5_ = 52.7  O_3_ = 128.3 | **Per 100,000 individuals:**  IHD: 12.7  Stroke: 10.2 | **Per 1000 individuals:**  IHD: 223.7  Stroke: 186.9 |
| Minghong Yao (2020) | Estimating health burden and economic loss attributable to short-term exposure to multiple air pollutants in China | China (338 cities) | 1.35 million | Male-female | Nd | PM_10_, SO_2_, NO_2_, CO, and O_3_ | PM_10_ = 79.73 SO_2_ = 18.14  NO_2_ = 30.23  CO = 0.96  O_3_ = 94.25 | CVD: 4,534,700 – 2,100,000 | CVD: 0.78 million |
| Wenyuan Yu (2020) | Burden of ischemic heart disease and stroke attributable to exposure to atmospheric PM_2.5_ in Hubei province, China | China (Hubei) | 57.24 million | Male-female | 30 - 69 | PM_2.5_ | 54.50 | Nd | **Per 1000 individuals:**  IHD: 18.92  Stroke: 36.84 |
| Wenjing Wu (2020) | Mortality burden attributable to long-term ambient PM_2.5_ exposure in China: using novel exposure-response functions with multiple exposure windows | China (national) | 1.94 million | Male-female | ≥25 | PM_2.5_ | 35.8 - 39.2 | Nd | IHD: 0.52 million  Stroke: 0.44 million |
| Prateek Saini (2020) | Cause and Age-specific Premature Mortality Attributable to PM_2.5_ Exposure: An Analysis for Million-Plus Indian Cities | India (29 cities) | 29 million | Male-female | 25 - ≥80 | PM_2.5_ | 27 - 143 | IHD: 85.56  Stroke: 35.75 | IHD: 66,100  Stroke: 25,500 |
| Dieyi Chen (2020) | Utilizing daily excessive concentration hours to estimate cardiovascular mortality and years of life lost attributable to fine particulate matter in Tehran, Iran | Iran (Tehran) | 106,180 | Male-female | 0 - ≥75 | PM_2.5_ | 34.7 | Nd | **Average daily mortality:**  CVD: 58.1  Stroke: 9.9  IHD: 18.1  SCD: 16.0 |
| Aiymgul Kerimray (2020) | Trends and health impacts of major urban air pollutants in Kazakhstan | Kazakhstan (21 cities) | 8,134 | Male-female | 25 - ≥85 | TSP, NO_2_, SO_2_, O_3_ | TSP = 157  NO_2_ = 51  SO_2_ = 29  O_3_ =41 | Nd | IHD: 4080  Stroke: 1613 |
| Lina Wang (2021) | Global burden of ischemic heart disease attributable to ambient PM_2.5_ pollution from 1990 to 2017 | Global  (195 countries) | Nd | Male-female | Nd | PM_2.5_ | Nd | **Per 100,000** **individuals:**  IHD: 14.45 - 12.46 | IHD: 544,040 – 977,140 |
| Qingfeng Ma (2021) | Temporal trend and attributable risk factors of stroke burden in China, 1990–2019: an analysis for the Global Burden of Disease Study 2019 | China (national) | 3.94 million | Male-female | 15 - ≥80 | PM_2.5_ | Nd | **Per 100,000 individuals:**  Stroke: 29.5  Ischemic stroke; 13.9  Intracerebral hemorrhage: 14.4  Subarachnoid hemorrhage: 1.3 | Stroke: 542,000  Ischemic stroke: 245,500  Intracerebral hemorrhage: 272,100  Subarachnoid hemorrhage: 24,400 |
| Jie Ban (2021) | Associations between short-term exposure to PM_2.5_ and stroke incidence and mortality in China: A case-crossover study and estimation of the burden | China  (10 counties) | 154,965 | Male-female | 0 - >75 | PM_2.5_ | 53.9 | Nd | Stroke: 23,018  Ischemic stroke: 7,342  Hemorrhagic stroke: 11,922 |
| Jie Li (2021) | The association between ozone and years of life lost from stroke, 2013-2017: a retrospective regression analysis in 48 major Chinese cities | China  (48 cities) | Nd | Male-female | <65 - ≥65 | O_3_ | 86.9 | Nd | Stroke: 2.15 million |
| Jie Li (2021) | Short-term effects of ambient nitrogen dioxide on years of life lost in 48 major Chinese cities, 2013-2017 | China  (48 cities) | 403 million | Male-female | 0 - ≥75 | NO_2_ | 39.7 | Nd | CVD: 53 |
| D. Rojas-Rueda (2021) | Ambient particulate matter burden of disease in the Kingdom of Saudi Arabia | Kingdom of Saudi Arabia (national) | 19,569 | Male-female | 1 - ≥95 | PM_2.5_ | 87.9 | **Per 100,000 individuals:**  IHD:  1990: 24  2010: 35  2017: 30  Stroke:  1990: 10  2010: 10  2017: 8 | IHD:  1990: 1401  2010: 3914  2017: 4634  Stroke:  1990: 554  2010: 986  2017: 1056 |
| Guijie Luan (2021) | Associations between ambient air pollution and years of life lost in Beijing | China (national) | 386,695 | Male-female | ≤65 - >65 | Nd | API = 84 | Nd | CVD: 185,360  IHD: 84,504  Stroke: 85,503 |
| Ming Liu (2021) | Recent trends in premature mortality and health disparities attributable to ambient PM_2.5_ exposure in China: 2005-2017 | China (national) | Nd | Male-female | ≤65 - >65 | PM_2.5_ | <5 - >105 | Nd | IHD: 580,000  Stoke: 560,000 |
| Maria D. Castillo (2021) | Estimating Intra-Urban Inequities in PM_2.5_-Attributable Health Impacts: A Case Study for Washington, DC | USA (Washington) | 627,656 | Male-female | 0 - 99 | PM_2.5_ | 10 - 17.1 | **Per 100,000 individuals:**  IHD: 7-58  Stroke: 0.5-3.75 | IHD: 90  Stroke: 10 |
| Sheng Zheng (2021) | Spatial Distribution of PM_2.5_-Related Premature Mortality in China | China (338 cities) | 1.55 million | Male-female | <5 - >25 | PM_2.5_ | 45.9 | Nd | IHD: 0.534 million  CEV: 0.525 million |
| Marcos Lorran Paranhos Leão (2021) | Health impact assessment of air pollutants during the COVID-19 pandemic in a Brazilian metropolis | Brazil (Recife) | 1,653,461 | Male-female | 15 - >65 | PM_10_, PM_2.5_ | PM_10_ = 15.5 - 22  PM_2.5_ = 7.33 -13 | Nd | CVD: 73 |
| Alen Juginović (2021) | Health impacts of air pollution exposure from 1990 to 2019 in 43 European countries | Europe  (43 countries) | Nd | Male-female | <5 - >70 | PM_2.5_ | 20.8 – 13.8 | **Per 100,000 individuals:**  IHD: 6.0  Stroke: 3.2 | IHD: 155,100  Stroke: 86,400 |
| Shaowei Sang (2022) | The global burden of disease attributable to ambient fine particulate matter in 204 countries and territories, 1990–2019: A systematic analysis of the Global Burden of Disease Study 2019 | Global  (204 countries) | 6,000,000 | Male-female | 0 – ≥95 | PM_2.5_ | Nd | **Per 100,000 individuals:**  CVD:  1990: 30.8  2019: 30.9  IHD:  1990: 16.8  2019: 16.6  Stroke:  1990: 14.0  2019: 14.3  Intracerebral hemorrhage Stroke:  1990: 6.0  2019:6.8  Ischemic stroke:  1990: 6.7  2019:6.6  Subarachnoid hemorrhage stroke:  1990: 1.2  2019: 0.8 | CVD:  1990: 607,580  2019: 2,475,400  IHD:  1990: 607,580  2019: 1,332,040  Stroke:  1990: 508,030  2019: 1,143,350  Intracerebral hemorrhage stroke:  1990: 232,750  2019: 558,540  Ischemic stroke:  1990: 225,610  2019: 515,960  Subarachnoid hemorrhage stroke:  1990: 49.67  2019: 68.85 |
| Yuzhi Xi (2022) | Association Between Long-term Ambient PM_2.5_ Exposure and Cardiovascular Outcomes Among US Hemodialysis Patients | United States (national) | 314,079 | Male-female | 63.6 | PM_2.5_ | 8.7 | Nd | CVD: 35,857 |
| Benjamin M. Varieur (2022) | Air Pollution, Political Corruption, and Cardiovascular Disease in the Former Soviet Republics | Soviet republics (Eastern Europe-Western Europe) | 178,000 | Male-female | Nd | PM_2.5_ | 12.4 - 20.0 | **CVD**  **Per 100,000 individuals:**  Eastern Europe: 65.44  Western Europe: 29.09 | CVD: 178,000 |
| Luisa Campos Caldeira Brant (2022) | Burden of Cardiovascular diseases attributable to risk factors in Brazil: data from the "Global Burden of Disease 2019" study | Brazil (national) | Nd | Male-female | Nd | PM_2.5_ | 2.4 - 5.9 | CVD:  1990: 55.1  2019: 13.4 | Nd |
| Hui Gan (2022) | Deaths and disability-adjusted life years burden attributed to air pollution in China, 1990–2019: Results from the global burden of disease study 2019 | China (national) | 3.75 million | Male-female | 0-≥70 | PM_2.5_, O_3_ | Nd | **Per 100,000 individuals:**  Stroke: 36.96  IHD: 26.41 | Nd |
| Myriam Tobollik (2022) | Burden of Disease Due to Ambient Particulate Matter in Germany—Explaining the Differences in the Available Estimates | Germany (national) | 15,600 | Male-female | <1 - ≥95 | PM_2.5_ | 13.7 - 10.8 | **Per 100,000 individuals:**  IHD: 11.07  Stroke: 2.97 | IHD: 6,977  Stroke: 1,871 |
| Philip J. Landrigan (2022) | A replicable strategy for mapping air pollution’s community‑level health impacts and catalyzing prevention | USA (Massachusetts) | 2,780 | Male-female | 0 - ≥65 | PM_2.5_ | 6.3 | Nd | Heart Disease: 1677  Stroke: 200 |
| Weiwei Wang (2023) | Time Trends in Ischemic Heart Disease Mortality Attributable to PM_2.5_ Exposure in Southeastern China from 1990 to 2019: An Age-Period-Cohort Analysis | China (Jiangsu) | 23,000 | Male-female | 25 - 94 | PM_2.5_ | Nd | **Per 100,000 individuals:**  IHD: 18.90-11.53 | IHD: 8,636 - 14,462 |
| Mengmeng Yan (2023) | State-level disparities in burden of ischemic heart diseases mortality attributable to ambient fine particulate matter in the United States, 1990–2019: Observational analysis for the Global Burden of Disease (2019) study | USA (National) | Nd | Male-female | Nd | PM_2.5_ | Nd | **Per 100,000 individuals:**  IHD: 8.4 | Nd |
| Mohamad Iqbal Mazeli (2023) | Cardiovascular, respiratory and all-cause (natural) health endpoint estimation using a spatial approach in Malaysia | Malaysia (national) | 2000: 23,494,900  2008: 27,567,600  2013: 30,213,700 | Male-female | 25 - ≥85 | PM_2.5_ | 2000 = 22  2008 = 18  2013 = 24 | IHD:  2000: 22,158  2008: 22,892  2013: 20,295  Stroke:  2000: 11,290  2008: 13,430  2013: 22,282 | Nd |
| Hugo Grisales-Romero (2023) | Local attributable burden disease to PM_2.5_ ambient air pollution in Medellín, Colombia, 2010–2016 | Colombia (Medellín) | 3,873 | Male-female | 0 - ≥80 | PM_2.5_ | 35.8 | **Per 100,000 individuals:**  IHD:  2010: 9.0  2011: 8.8  2012: 9.7  2013: 9.4  2014: 9.5  2015: 8.6  2016: 9.2  CVD:  2010: 1.5  2011: 1.2  2012: 1.5  2013: 1.5  2014: 1.5  2015: 1.6  2016:1.3 | IHD:  2010: 210  2011: 238  2012: 266  2013: 262  2014: 268  2015: 250  2016: 263  CVD:  2010: 36  2011: 29  2012: 35  2013: 35  2014: 37  2015: 38  2016:33 |
| Shiyu Zhang (2023) | Exposure to Air Pollution during Pre-Hypertension and Subsequent Hypertension, Cardiovascular Disease ,and Death: A Trajectory Analysis of the UK Biobank Cohort | UK (national) | 168,010 | Male-female | 54.2 | PM_2.5_, PM_10_, NO_2_, Nox | PM_2.5_= 9.95 -10.06  PM_10_= 19.23-19.41  NO_2_= 29.00-29:93  NOx= 43.54 -44.93 | Hypertension: 2.66%  CVD: 16.63% | CVD: 7,643 |
| Nd – Not defined  IHD – Ischemic Heart Disease  CVD – Cardiovascular Disease  CHD – Coronary Heart Disease  CEV – Cerebrovascular Disease  AMI – Acute Myocardial Infarction  HHD – Hypertensive Heart Disease  SCD – Sudden Cardiac Death | | | | | | | | | |
